# Supplementary material for: The Ceramide Synthase Subunit Lac1 Regulates Cell Growth and Size in Fission Yeast
Source: Int J Mol Sci. 2021 Dec 28;23(1):303. doi: 10.3390/ijms23010303 (PMC8745161; doi:10.3390/ijms23010303)
Supplement: Supplementary file 1 [file ijms-23-00303-s001.zip › ijms-1504827-SI.pdf]

Supplementary Materials

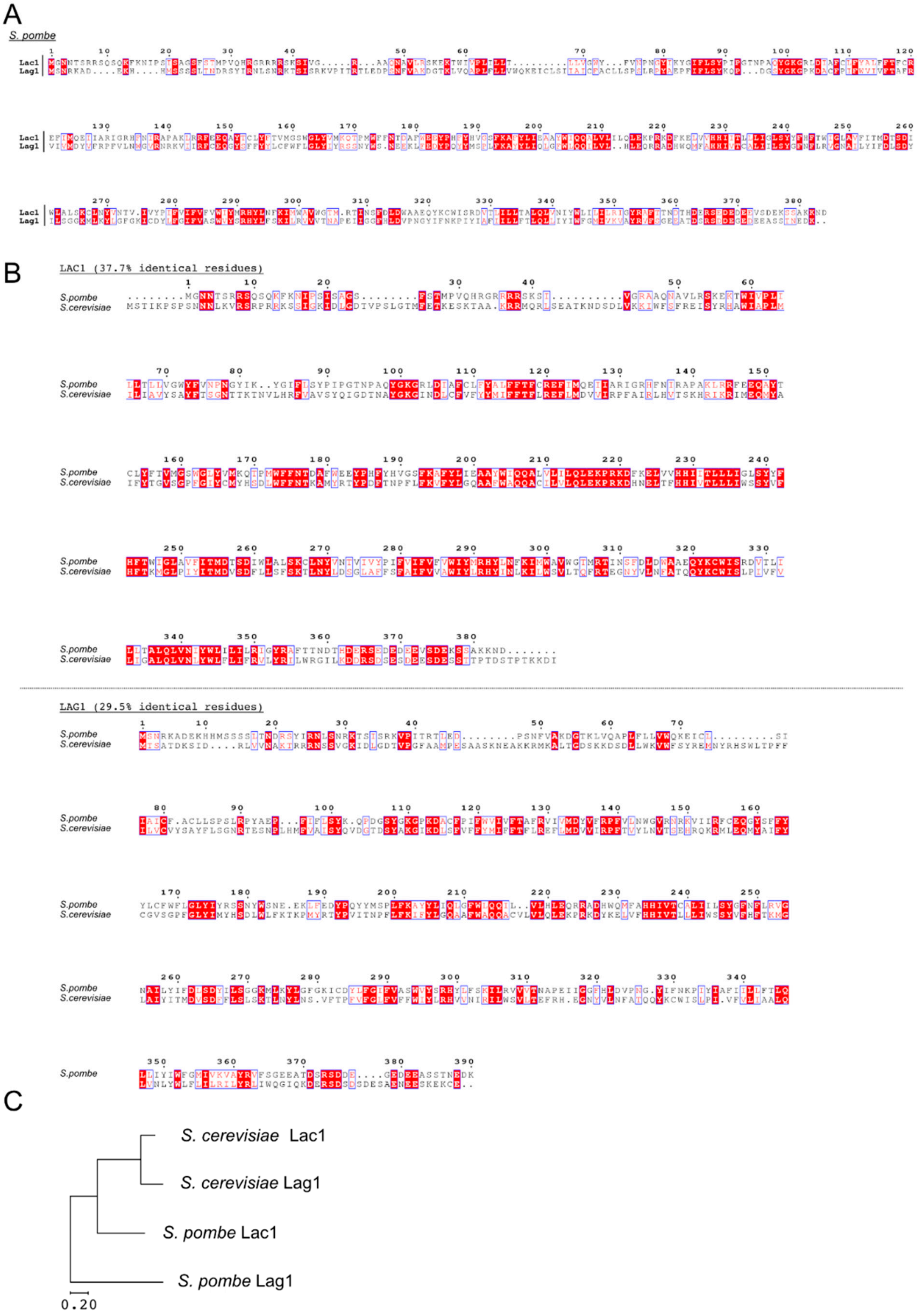

Figure S1. (**A**) Upper panel: sequence alignment between Lac1 subunits in fission and budding yeast. Lower panel: sequence alignment between the Lag1 subunits in fission and budding yeast. (**B**) Sequence alignment between ceramide synthase subunits in *S. pombe*, Lac1 and Lag1. White characters on red boxes denote identical residues, and red characters on white boxes denote conserved residues. (**C**) A phylogenetic tree using maximum-likelihood statistical method. Horizontal lines are proportional to the substitution rate. The bar represents 0.20 changes per amino acid. In panels A and B, white characters on red boxes denote identical residues, and red characters on white boxes denote conserved residues.

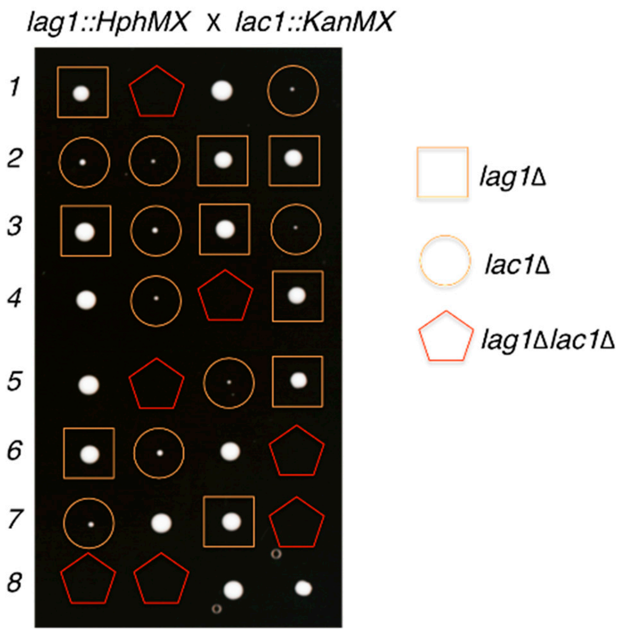

Figure S2. Deletion of Lac1 and Lag1 in *S. pombe* is lethal. Tetrad analysis of the indicated strains in Yes media at 25 °C.
